# Utilization of genetics services in the diagnosis of hearing loss in newborns in the state of Ohio

**DOI:** 10.1007/s12687-025-00816-0

**Published:** 2025-07-09

**Authors:** Cara L. Barnett, Prashant Malhotra, Allyson VanHorn, Boriana Zaharieva, John Myers, William J. Riggs, Elizabeth Jordan

**Affiliations:** 1 Division of Human Genetics, Department of Internal Medicine, The Ohio State University, Columbus, OH USA; 2 Department of Otolaryngology and the Hearing Program, Nationwide Children’s Hospital, Columbus, OH USA; 3 Center for Biostatistics, College of Medicine, The Ohio State University Wexner Medical Center, Columbus, OH USA; 4 Child and Specialty Health Services, The Ohio Department of Health, Columbus, OH USA; 5 Regeneron Pharmaceuticals, Tarrytown, NY USA

**Keywords:** Newborn hearing screening, Hearing loss, Genetic testing, Genetic services

## Abstract

**Introduction:**

In 50–60% of confirmed congenital hearing loss (HL) diagnoses, the etiology is genetic. The importance of a genetic evaluation for HL is recognized by several national organizations in the United States. This study aimed to evaluate provider practice patterns, beliefs, and knowledge of the role of genetics in the medical diagnosis of HL and assess parent experience and knowledge regarding the role of genetics in the diagnostic process.

**Methods:**

Two surveys were designed using published guidance on optimal care of newborns with HL. Participants included providers (otolaryngologists (ENT) and audiologists) and parents of a newborn with confirmed HL in the state of Ohio from 2017 to 2018.

**Results:**

95 providers (14 ENT; 81 audiologist) and 39 parent responses were included in the analysis. Only 51% of providers refer for a genetics evaluation (*n* = 49), and less than 10% order genetic testing (*n* = 9). However, 96% of providers believe families should be presented with the opportunity to pursue a genetics evaluation. In this study, only 46% (*n* = 18) of parents reported that they were referred to genetics, and 36% (*n* = 14) reported that their child had genetic testing for HL. For parentss whose child did not have a genetic evaluation, 53% (*n* = 17/32) were very likely or likely, 25% (*n* = 8/32) were unsure, and 22% (*n* = 7/32) were very unlikely or unlikely to pursue an evaluation.

**Conclusion:**

There is inconsistent implementation of guideline directed care for genetic services for HL. As opportunities for gene therapies for HL advance, there is a need to expand access to genetic evaluation for HL.

**Supplementary Information:**

The online version contains supplementary material available at 10.1007/s12687-025-00816-0.

## Introduction

Congenital hearing loss (HL) is a common condition detected at birth in developed countries, with a reported prevalence of 1.8 in 1,000 newborns screened (CDC 2020). Studies have repeatedly demonstrated that the earlier a HL diagnosis is made and intervention is initiated, the more likely a child will develop language, in addition to improved communicative, cognitive, social-emotional and literacy skills (Yoshinaga-Itano et al. 1998; Yoshinaga-Itano 2004; Larsen et al. 2012; Linden Phillips et al. 2012; Tomblin et al. 2014; Luckner and Movahedazarhouligh 2019). Accordingly, newborn screening for HL became a major public health effort in 1993, becoming a part of the newborn screening protocol on August 1, 2002 in the state of Ohio (Ohio Revised Code 3701.504).

Genetic and environmental (i.e., acquired) causes of HL have been described, with the most common environmental cause being congenital cytomegalovirus infection. An estimated 50–60% of HL is estimated to be due to genetic cause (Morton and Nance 2006). Genetic testing for HL is broadly available and the current diagnostic yield of panel-based genetic testing is ~ 40% (Shearer and Smith 2015). Clinical genetic evaluation, including genetic testing and counseling, is recognized as a key component of the diagnostic process for HL in newborns by several professional organizations, including the Joint Committee on Infant Hearing (JCIH), the American College of Medical Genetics (ACMG), the American Academy of Pediatrics (AAP), the American Speech-Language-Hearing Association (ASHA), and The International Pediatric Otolaryngology Group (IPOG) (Table 1), with all organizations agreeing that infants with HL should be offered a genetic evaluation as part of the diagnosis (JCIH 2007; Harlor and Bower 2009; Alford 2014; Mercer 2015; Liming et al. 2016; JCIH 2019; ACMG 2024).

**Table 1:** Genetic testing guidelines and recommendations for the diagnosis of newborn hearing loss

Topic		Members of the multidisciplinary team	Age care should be received	Hearing provider* role in genetic risk assessment	First-tier genetic testing
Organization	Publication Year	Recommendations			
Joint Committee on Infant Hearing (JCIH)	2007	• Pediatrician/Primary Care Provider • Otolaryngologist • Genetics • Ophthalmologist • Developmental pediatrics • EHI/FSS^+^ • Social work	• 3 months	• Obtain family history • Refer for genetic evaluation	
American Academy of Pediatrics (AAP) Clinical Report	2009	• Otolaryngologist • Audiologist • SLP^++^ • Genetics • EHI/FSS^+^			• *GJB2* and *GJB6* sequencing +/- del/dup^†^
American College of Medical Genetics (ACMG) ACT Sheet	2010 and 2024	• Multidisciplinary team • Clinical geneticist or genetic counselor	• 3 months • Early intervention by 6 months		
ACMG	2014	• Otolaryngologist • Clinical geneticist • Genetic counselors • Audiologists • SLP^++^ • EHI/FSS^+^ • Other appropriate specialists		• Obtain family history • Refer for genetic evaluation	• *GJB2* and *GJB6* sequencing +/- del/dup^†^
American Speech-Language-Hearing Association (ASHA)	2015			• Refer for genetic evaluation	
International Pediatric OOtolaryngology Group (IPOG)	2016			• Order first tier genetic testing • Consider referral for genetics evaluation	• Multi-gene HL panel • If panel unavailable: *GJB2* and *GJB6* sequencing +/-del/dup^†^
Joint Committee on Infant Hearing (JCIH)^††^	2019	• Pediatrician/Primary Care Provider • Otolaryngologist • Genetics • Ophthalmologist • Developmental pediatrics • EHI/FSS^+^ • Social work	• 2 months	• Obtain family history • Consider *GJB2/GJB6* testing • Consider electrocardiogram • Refer for genetic evaluation	• *GJB2/GJB6* testing with acknowledgement of multi-gene HL panel as most comprehensive option
ACMG^††^	2022	• Otolaryngologist • Clinical geneticist • Genetic Counselors • Audiologist • SLP^++^ • EHI/FSS^+^ • Other appropriate specialists		• Obtain a family history • Perform a physical exam for syndromic features • Order multi-gene HL panel testing • Refer for genetic evaluation and multidisciplinary care	• Multi-gene HL panel with consideration of ES or GS if negative results^#^

*Hearing provider refers to otolaryngologists and/or audiologists

+ Early Hearing Intervention and Family Support Services (EHI/FSS)

++ Speech Language Pathologist

† +/- del/dup refers to with or without deletion/duplication analysis

†† JCIH 2019 and ACMG 2022 guidelines were not available and therefore not included in initial development of study documents including the provider and parent surveys

# ES is exome sequencing, GS is genome sequencing

Despite national guidance and universal newborn screening for HL mandated in many states, genetics is under-utilized in this population, with a recent study regarding family experiences across the United States reporting that nearly half of children with HL had genetic testing. For those who did not undergo genetic testing, the largest reason this was not pursued is because it was never offered (Cejas et al. 2024). The reasons for this practice deficit are unknown and may vary due to local and regional process and resource differences. We aimed to assess provider utilization of genetics services in the management of HL in the state of Ohio and to evaluate parent/family experience with clinical genetics in the diagnosis of their child’s HL. A combined understanding of provider practice patterns and parent experiences will enable the development of targeted strategies designed to maximize accessibility of genetics services to the HL population.

## Materials and methods

### Recruitment

Two populations were invited to participate: HL providers (otorhinolaryngology (ENTs)) and audiologists (M.A./M.S. or Au.D.) and parents/guardians of children with HL. Providers were eligible if they were practicing ENTs or audiologists in Ohio working primarily with pediatric or newborn populations. Parents/guardians were eligible if they had a child born in Ohio between January 1, 2017, and December 31, 2018, who had a clinical diagnosis of HL (e.g., mild, moderate, severe, profound) not deemed to be due to an acquired cause identified by the Ohio Department of Health (ODH) Infant Hearing Program (IHP) program. These years were selected as the most recent birth cohorts for which address data was available at the time of survey distribution (August-December 2019). Providers and patients self-reported eligibility to participate in the study. Provider recruitment took place by e-mail through personal and professional networks. Parents/guardians were recruited by mailed packet in collaboration with ODH.

### Procedures

Survey instruments for each population (provider survey and parent/guardian survey) were designed to assess practice patterns and patient experience in relation to the recommendations from AAP, JCIH, ACMG, AJA, and IPOG (Table 1). A validated Genetics Knowledge Measure was used to assess parent genetic knowledge (Fitzgerald-Butt et al. 2015). Both surveys included an open-ended question to allow for additional comments. Providers were queried by electronic survey using Qualtrics. Parents/guardians were provided the option for either an electronic survey or paper survey format. As an incentive, both parent and provider participants were invited to enter a drawing for gift cards.

### Analysis

Descriptive statistical analysis was performed. Generalized comparisons were made between provider reported practice and parent perceived experience. Responses were extracted into SAS 9.4 for data cleaning, categorization, and analysis. For the parent survey (Supplemental Material - Appendix 2), Sect. 3, Genetics Knowledge Measure, a Mann-Whitney U Test was used to determine statistical significance. For Sect. 4, question 1, parent knowledge of HL, a Fisher’s exact test was used in contingency table analysis. For Sect. 5 analyses, experience with genetics in child’s HL diagnosis, Fisher’s exact tests were used to assess statistical significance. For the provider survey (Supplemental Material - Appendix 1), Sect. 1, question 6, to assess the relationship between years in practice and training, a Fisher’s exact test was used with a contingency table, with a logistic regression used to adjust for ENT versus audiologist. For overall analysis of Sect. 3 of the provider survey, the Wilcoxon rank-sum test was used as scores were not normally distributed.

## Results

The study received multi-institutional review board approval from The Ohio State University, ODH, and Nationwide Children’s Hospital. Informed consent was obtained. After being shown the informed consent document, participants indicated their consent to participate by continuing with the survey.

### Provider survey

A total of 113 responses were received from providers and 95 total were included in analysis. Eighteen were excluded due to incomplete survey or provider type not specified. Most providers were white (*n* = 90, 95%), female (*n* = 81, 85%), and audiologists (*n* = 78, 82%) (Table 2). Half (*n* = 48, 51%) of providers had participated in a continuing education event regarding the genetics of HL, and most providers are interested in additional continuing education regarding the genetics of HL (*n* = 82, 93%). Correct responses to general HL genetics knowledge questions varied, with best performance regarding inheritance pattern questions, but lack of appropriate estimation of recurrence risk for the siblings of a child with connexin HL.

**Table 2:** Provider demographics

Sex	Frequency (%)
Male	14 (15)
Female	81 (85)
**Race/ethnicity**	**Frequency (%)**
White	90 (95)
Black or African-American	2 (2)
Hispanic	1 (1)
Other	2 (2)
**Years in Clinical Practice**	**Frequency (%)**
0–4 years	22 (23)
5–9 years	19 (20)
10–19 years	30 (32)
More than 20 years	23 (25)
**Education**	**Frequency (%)**
Master’s degree	78 (82)
Professional degree (MD, PhD, JD, DO, DDS etc.)	17 (18)

Demographics as self-reported by provider survey respondents including otolaryngologists and audiologists practicing primarily in pediatrics in the state of Ohio. The most common respondents were female audiologists with a master’s degree and a diverse range of years in practice

**Fig. 1:**
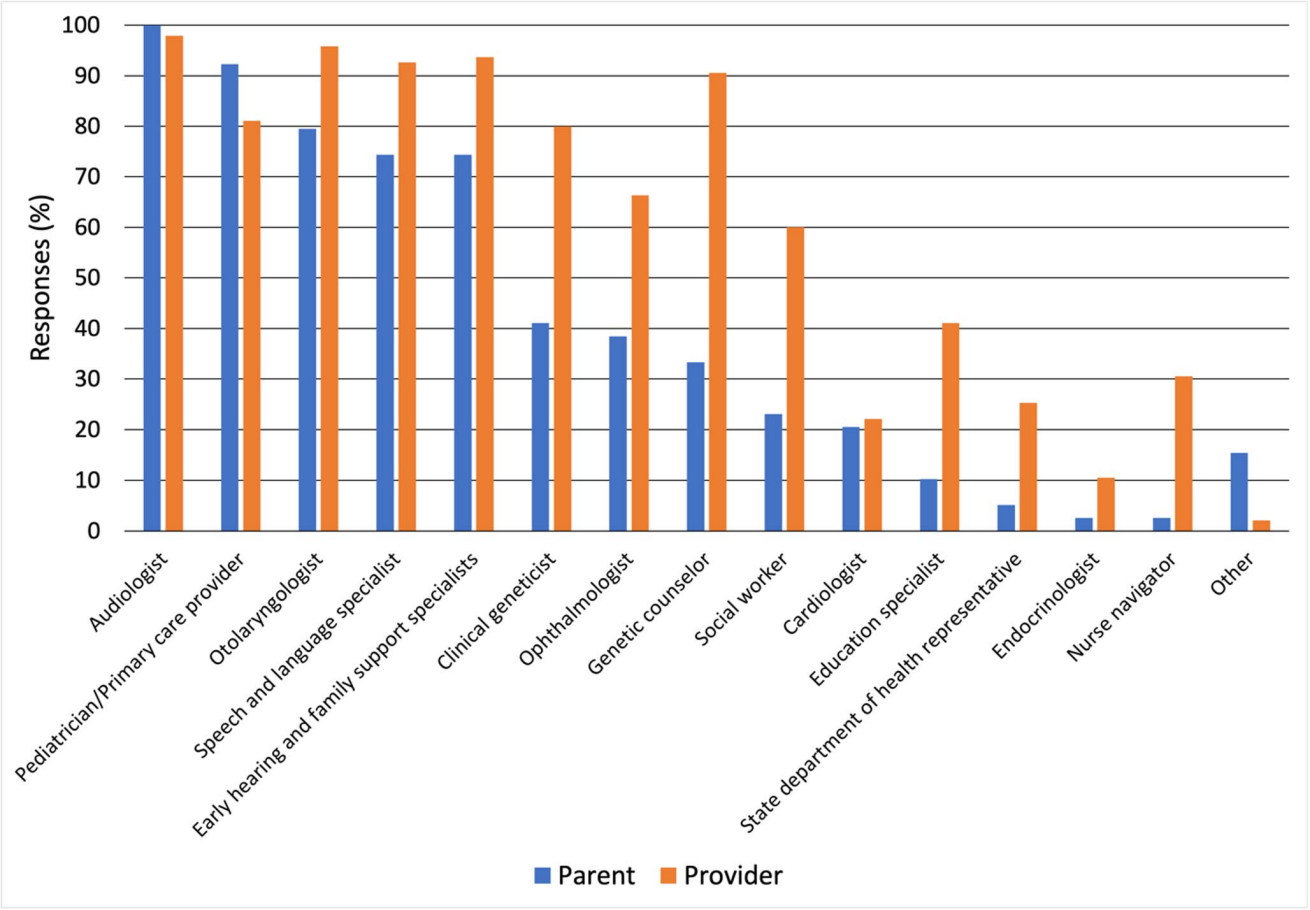
Providers involved in the multidisciplinary care team for HL as reported by Proverds and Parents of infants with HL

All providers reported that the medical home for newborns with hearing thresholds outside the typical range should have a multidisciplinary team involved (Fig. 1). A key aspect of this care involves defining responsibilities within the team, particularly regarding the discussion of genetics in HL. When asked which provider in the multidisciplinary team is most responsible for informing a family about the role of genetics in HL, 59% of providers reported the ENT is most responsible. While no ENTs reported this as the primary responsibility of the audiologist, 35% (*n* = 28) of audiologists self-reported discussing genetics as the audiologist’s primary responsibility.

Approximately 96% (*n* = 85) of provider participants strongly believe or believe that families should be presented with the opportunity to pursue a genetic evaluation but variability in practice patterns was also reported. While 85% (*n* = 81) of providers frequently obtain a family history, only 52% (*n* = 49) refer for a genetics evaluation, and fewer than 10% (*n* = 9) order genetic testing. Specifically considering ENT practice patterns, less than half of ENTs reported ordering connexin (*GJB2*/*GJB6*) gene testing, and no ENTs reported ordering comprehensive multi-gene panels for HL (Table 3).

**Table 3:** Provider survey response: which actions are within your primary responsibility in clinical practice??

What actions are within your primary responsibilities in clinical practice?	ENT	Audiologist	Combined
	Frequency (%)	Frequency (%)	Frequency (%)
Obtaining a family history	10 (71)	71 (88)	81 (85)
Referring for a genetics evaluation	13 (93)	36 (44)	49 (52)
Facilitating shared decision making	13 (93)	24 (30)	37 (39)
Performing physical examination for syndromic features	11 (79)	7 (9)	18 (19)
Pre-test counseling for genetic testing	4 (29)	7 (9)	11 (12)
Ordering connexin (GJB2 and GJB6) testing	7 (50)	1 (1)	8 (8)
Post-test counseling for genetic testing	1 (7)	5 (6)	6 (6)
Genetic testing for family members	1 (7)	2 (3)	3 (3)
Genetic testing results disclosure to the family	2 (14)	0 (0)	2 (2)
Interpretation of genetic test results	2 (14)	0 (0)	2 (2)
Ordering comprehensive multi-gene panels for hearing loss	0 (0)	1 (1)	1 (1)

A multi-response question was used to assess provider practice patterns, with data analyzed by provider type and combined across all providers. The most frequently reported responsibilities differed between groups: ENTs commonly cited referring for genetics and facilitating shared decision-making, while audiologists emphasized obtaining a family history and referring for genetics

About three quarters (76%, *n* = 68) of providers strongly believe or believe that the families for whom they care for would be interested in a genetics evaluation. While providers generally follow published guidance, variability exists in application. Not all providers consistently discuss genetics or order genetic testing in clinical practice.

### Parent/guardian responses

A total of 45 surveys were collected from participants who were parents of infants with HL. Six (6) surveys were excluded from analysis (4 for ineligibility; 2 surveys with only demographic information provided). Of the 39 parent responses included in the analysis, the majority were white (*n* = 35, 90%), female (*n* = 32, 82%), and between 30 and 39 years old (*n* = 26, 67%) (Table 4). All participants were a biological parent of their child with HL. When parents were asked to report the type, severity, and laterality of their child’s HL diagnosis, many did not know or did not respond. The overall mean Genetic Knowledge score was 15.89 out of 18. There was no statistical significance between knowledge of HL diagnosis and parental education level.

**Table 4:** Parent demographics

Sex	Frequency (%)
Male	7 (18)
Female	32 (82)
**Race/ethnicity**	**Frequency (%)**
White	35 (90)
Black or African-American	1 (3)
Hispanic	3 (8)
Other	0 (0)
**Age**	**Frequency (%)**
18–25	1 (3)
26–29	9 (23)
30–39	26 (67)
40–49	3 (8)
**Education**	**Frequency (%)**
Some high school	1 (3)
High school graduate (diploma or the equivalent (GED)	3 (8)
Some college credit	6 (15)
Bachelor’s degree	12 (31)
Associate degree	6 (15)
Master’s degree	8 (21)
Professional degree (MD, PhD, JD, DO, DDS etc.)	3 (8)
**Income (** ***n*** ** = 37)**	**Frequency (%)**
Less than $25,000	2 (5)
$25,000 to $49,999	4 (11)
$50,000 to $99,999	19 (51)
$100,000 to $149,999	6 (16)
$150,000 or more	6 (16)
**Insurance**	**Frequency (%)**
Private/commercial insurance	29 (74)
Medicaid insurance	8 (21)
Other	2 (5)

Self-reported demographics of parent survey respondents, including parents of newborns diagnosed with hearing loss through the Ohio Infant Hearing Program between January 2017 and December 2018. Most respondents were white females aged 30–39 years, holding a bachelor’s degree or higher, with an income above $50,000, and covered by private insurance. Percentages may not sum to 100% due to rounding

Families reported a multidisciplinary team of providers and specialists involved in the care of their children (Fig. 1). Less than half (*n* = 18, 47%) of parents reported their child was seen by all providers within three months, while 34% (*n* = 13) were seen from age 4–6 months and 18% (*n* = 7) from age 8–18 months. When asked which providers discussed the role of genetics in HL with the family, ENTs were most common (*n* = 16, 57%), followed by the audiologist (*n* = 9, 32%) (Table 5). Parents report a wide variety of information discussed regarding the benefits and drawbacks of genetic testing. Eighteen (46%) families reported that they were referred for a genetic evaluation, and 14 (37%) reported that their child had genetic testing for HL. Regarding genetic diagnoses: three reported *GJB2* pathogenic variants (21%), five reported syndromic HL, and five families no genetic cause was determined. Seventeen families (45%) without prior genetic testing were very likely or likely to pursue a genetics evaluation.

**Table 5:** Parental report of which provider(s) discussed the possible role of genetics in their child's diagnosis of HL

Providers who discussed a possible genetic cause for their child’s hearing loss	Frequency (%)
Otolaryngologist	16 (57)
Audiologist	9 (32)
Clinical Geneticist	8 (28)
Genetic counselor	4 (14)
Pediatrician/Primary Care Provider	3 (11)
Speech and language specialist	2 (7)
Early hearing intervention and family support specialists	1 (4)
Ophthalmologist	0 (0)
Social worker	0 (0)
State Department of Health Representative	0 (0)
Cardiologist	0 (0)
Endocrinologist	0 (0)
Nurse Navigator	0 (0)
Education Specialist	0 (0)
Other	4 (14)
**Provider that referred their child for genetic HL evaluation**	**Frequency (%)**
Otolaryngologist	6 (33)
Pediatrician/Primary Care Provider	4 (22)
Audiologist	1 (6)
Other	6 (33)
I do not remember who referred my child to see a genetics provider	1 (6)

A parent survey question assessed their recall of the provider who discussed genetic testing and who referred their child to genetics. Most parents reported that the otolaryngologist discussed genetic testing, followed by the audiologist. Among parents whose child was referred to genetics, the otolaryngologist was the most common referrer, followed by “other” (multiple providers listed in free-response answers) and the pediatrician/primary care provider

## Discussion

This study sought to evaluate the use of existing guidance for the genetic evaluation of HL in Ohio through surveying parents of children with HL and providers involved in the diagnosis and care of HL. The first effort to evaluate this in the state of Ohio, these results demonstrated inconsistency with provider practice and perceived responsibilities, and variable parent experiences in receiving genetic HL evaluation. These findings underscore important deficits and opportunities for both provider and community education in this area.

Many parents did not provide the type and severity of their child’s HL. Prior literature has demonstrated variable recall of diagnostic hearing results, more commonly only reporting knowledge of final diagnosis but poor understanding of the details of the audiogram and hearing mechanism (Watermeyer et al. 2012). This may be due, in part, to emotional responses such as grief, worry, and sadness to a new HL diagnosis that may impact one’s memory of fine details (Gilliver et al. 2013). Variable knowledge may also be due to health literacy (Gilbey 2010; Minchom et al. 2013). The timing and modality used to relay diagnostic information and strategic timing may also have an impact. During the time of this study, it was not required of Ohio HL providers to provide the diagnostic hearing evaluation results in writing. OAC 3701-40-08 was implemented on December 1, 2019, and hearing screening providers are now required to provide the results of the hearing evaluation in writing to parents/caregivers upon completion of the diagnostic evaluation. Future studies are needed to assess impact of this new documentation procedure.

Several participants commented on the timing of their child’s evaluation and their perceived impact of this timing. Participants 7 and 23 emphasized the importance of early detection and treatment of HL in an open response survey question: “*We are thankful for newborn screening because we were able to get him aided before he was three months old*” (Participant 7) and “*We are very thankful it was diagnosed early so we could get hearing aids and not lose any time to learn language*” (Participant 23.) In contrast, Participant 38 statesd, “*the way it was presented to me was that it was not something needed immediately so I deemed it not important right now.*” These contrasting Participant reflections suggest a need for providers to evaluate patients individually and provide flexibility in their plan for medical management. A variety of personal and medical factors can influence parental decision making, especially regarding genetic testing, and thoughtful interactions with the medical team are needed to support shared decision making.

The JCIH, AAP, and ACMG recommend that a multidisciplinary care model of HL include a referral for a clinical genetics evaluation (Table 1) (ACMG 2024; JCIH 2019, 2007; Li et al. 2022; Alford et al. 2014a, b; Harlor and Bower 2009). Less than half of participating parents reported receiving complete multidisciplinary care within three months, with three-quarters of affected children seeing all members of the multidisciplinary team by age six months. Further, less than half of parents (41%) interacted with genetics, despite 91% of providers indicating that genetics should be part of the care team (Fig. 1). This may be due to a lack of referrals from care providers, or lack of parental awareness of the referral and availability of genetics services. Lesperance et al. found that the optimization of guideline implementation is dependent upon consideration of the parent/family, informed decision making, and flexibility in approach and timing of the genetics evaluation (Lesperance et al. 2017). This survey was distributed between August and December 2019 to families of children born in 2017–2018, all affected children were three years of age or younger at the time of participation. As a result, this study captured family experiences relatively early in the post-diagnosis trajectory. Genetic testing within a few months after birth may offer clinical and reproductive benefits, including informing etiology, guiding medical management, and clarifying recurrence risk. However, we also recognize this cross-sectional approach and sample time frame as a limitation, as it is also possible that families may have pursued testing as their children at later ages than would have been captured by this study, albeit outside the recommended genetic testing time window according to US professional organizations.

A collaborative approach involving the primary care provider, ENT, ophthalmologist, developmental pediatrics, early hearing support specialists, social work, and genetics is recommended by JCIH (JCIH 2019). The recently updated ACMG clinical evaluation and diagnosis of newborn HL guidelines highlight the role of the genetic counselor and recommend all newborn HL patients receive post-test genetic counseling, regardless of testing result (Li et al. 2022). However, a barrier to this care model is currently limited size of the clinical genetics’ workforce, as recognized by The American Board of Medical Genetics and Genomics (Jenkins et al. 2021; Maiese et al. 2019). Novel service delivery models will be needed to accommodate access to genetics services for diverse patient populations, including HL (Raspa et al. 2021).

 Parental concerns about the cost of genetic testing was a commonly cited drawback. A recent study evaluating parental perceptions and experience of genetic testing for HL also extends this finding in a survey of 146 parents of children with HL, with less than half (48%) of participant’s children received genetic testing; cost was cited as a reason for not having testing in this study, in addition to being unaware of recommendation, not interested, time, fear of results, and testing not being offered (Cejas et al. 2024 ). Accordingly, families with a household income over $100,000 were more likely to pursue genetic testing ( *p*  = 0.044, Fisher’s Exact Test). This suggests that lower income may be a barrier to receiving genetic testing, and indirectly to receiving genetics services. Participant 2 commented that, “ *It would be nice if this was covered by insurance so we could test her genetics to see how this will impact her*, * if any other issues will arise*, * and what her future of a family would look like. However*, * the testing is not affordable so we will continue to focus on her treatments and whatever other issues arise.* ” A confirmed genetic diagnosis may, in some cases, lead to additional medical evaluations or surveillance due to associated health risks. For example, in Jervell and Lange-Nielsen syndrome, which includes bilateral sensorineural HL and a predisposition to Long QT syndrome, a genetic diagnosis will prompt a cardiac evaluation. Genetic diagnosis also has potential to reduce the need for ongoing diagnostic testing by clarifying the etiology early in the care pathway, potentially limiting long-term costs of the diagnostic journey for patients and families. Further, with a future of precision therapies including gene-specific treatments on the horizon for HL, diagnosing genetic etiology will be the gateway to access novel treatment approaches as they enter into clinical care. With such care options emerging, it is not surprising that cost has been shown to also be a provider concern, with another investigation reporting that ENTs voiced insurance barriers as preventing them from ordering genetic testing (Heyward et al. 2023 ).

The AAP and IPOG guidance state that ENTs should consider ordering connexin gene testing for HL, while the updated ACMG guidelines recommend a broadere HL gene panel for all newborns with HL (Harlor and Bower 2009; Liming et al. 2016; Li et al. 2022). Half of ENTs in this study reported ordering connexin gene testing as one of their primary responsibilities, while only 36% reported routinely ordering connexin genetic testing in clinical practice. No ENTs in this study reported ordering comprehensive HL gene panels. While this study did not directly evaluate reasons for lack of ENT uptake of genetic testing, an open-ended response was provided by a parent Participant that “*Genetic testing does not change the treatment. Hearing loss isn’t something we need to figure out or cure. Doctors push this testing for their own benefits/reasons. Even with testing hearing loss becoming more severe still isn’t predictable*”. This perspective underscores the importance of shared decision making in the genetic evaluation of HL. Genetic counselors are trained to facilitate the genetic testing decision making process, including both educational and psychological support as a part of this consideration, which is why guidelines recommend this service as a part of the HL care team. However, a recent study of parents who pursued genetic testing for their child with HL found that 55% did not receive counseling before testing, and only 42% received genetic counseling afterward. The study team noted that their data was consistent with prior research, which showed that parents who received either pre-test or post-test counseling reported a more positive experience with the genetic testing process (Cejas et al. 2024). Cultural identity in the HL community influences perspectives on genetic testing, highlighting the importance of informed decision-making that respects patient and family values.

An opportunity for comprehensive genetic evaluation of HL is essential to ensure accurate and timely diagnosis and personalized care planning. Emerging gene therapies for genetic forms of HL highlight the potential for personalized care, with a definitive genetic diagnosis being required for eligibility. Many different therapies are in development including gene replacement therapies via adeno-associated viral (AAV) vectors, antisense oligonucleotides, small molecules, and CRISPR-based gene editing (Omichi et al. 2019; Leclère et al. 2024). Gene replacement therapy using AAV for *OTOF*-related auditory neuropathy spectrum disorder (DFNB9) has shown early safety and efficacy in clinical trials in China, with ongoing studies worldwide, including in the United States (Lv et al. 2024; Wang et al. 2024). These rapidly evolving precision medicine treatments for HL emphasize the critical need for early genetic diagnosis to ensure access to emerging therapies (Shearer 2024).

There are limitations to this study. Some items analyzed in the survey instrument were not validated, we were limited by asmall sample size, and the study was only conducted in Ohio; Therefore, the reported results are not generalizable. In addition, both participant groups were comprised of mostly metropolitan/suburban, white, middle-class females. These data are crossectional and were collected within the first three years of life of the affected children, therefore it is possible genetic testing may have been performed at a later age, albeit outside of the recommended time window for genetic diagnosis according to current guidance. There is also potential ascertainment bias, with parents and providers with interest in the genetics of HL more likely to participate, in which case these utilization patterns reported may be overestimates. Additionally, the study materials were created and study completed prior to the release of the JCIH 2019 update and the ACMG 2022 update. The 2022 ACMG update includes a recommendation change to order a multi-gene HL panel, rather than *GJB2/GJB6* gene sequencing, for initial evaluation. While our study did not directly assess opinions regarding targeted gene testing versus multi-gene HL panels in providers, we can anticipate an updated survey may identify a broader movement to multi-gene HL panels.

## Conclusion

Collaborative, multidisciplinary team-based health care between the state department of health, providers, and patients is key to providing optimal care and support in HL. State-specific strategic initiatives could help increase access to genetic evaluations for families, thereby allowing timely access to genetic diagnosis supporting precision care approaches for HL.

## Electronic supplementary material

Below is the link to the electronic supplementary material.

Supplementary Material 1

## Data Availability

No datasets were generated or analysed during the current study.
