# Supplementary material for: Utilization of genetics services in the diagnosis of hearing loss in newborns in the state of Ohio
Source: J Community Genet. 2025 Jul 9;16(5):603–13. doi: 10.1007/s12687-025-00816-0 (PMC12401789; doi:10.1007/s12687-025-00816-0)
Supplement: Supplementary file 1 — Supplementary Material 1 [file 12687_2025_816_MOESM1_ESM.docx]

| **Genetics Knowledge Questions** | | **Frequency (%)** |
| --- | --- | --- |
| Percentage of Non-syndromic Hearing Loss Estimated to be Genetic | | 86 (51.9) |
| Most Common Genetic Mechanism for the Inheritance of Non-syndromic Hearing Loss | Autosomal Dominant | 11 (12.79) |
|  | Autosomal Recessive | 67 (77.91) |
|  | X-linked | 3 (3.49) |
|  | Mitochondrial | 4 (4.65) |
|  | Polygenic | 1 (1.16) |
| Risk for parents with a child with connexin related hearing loss to have another child with hearing loss  *Open ended, sliding scale*  (N=86) | | Average: 34.7% (SD: 16.2) |
| Gene responsible for the most common genetic form of non-syndromic hearing loss | GJB2 | 64 (75.29) |
| **Who are the healthcare providers on the primary multidisciplinary care team?** | | **Frequency (%)** |
| Pediatric Audiologists | | 93 (97.89) |
| Otolaryngologists | | 91 (95.79) |
| Early hearing intervention specialist | | 89 (93.68) |
| Speech and language specialists | | 88 (92.63) |
| Genetic counselors | | 86 (90.53) |
| Pediatrician/Primary Care Provider | | 77 (81.05) |
| Clinical geneticists | | 76 (80.00) |
| Ophthalmologists | | 63 (66.31) |
| Home-based family support | | 59 (62.11) |
| Social workers | | 57 (60.00) |
| Education Specialists | | 39 (41.05) |
| Nurse Navigator | | 29 (30.53) |
| ODH Rep | | 24 (25.26) |
| Cardiologists | | 21 (22.11) |
| Endocrinologists | | 10 (10.53) |
| Optometrists | | 6 (6.32) |
| Other | | 2 (2.11) |
| **Which healthcare provider is most responsible for informing family about role of genetics in hearing loss?** | | **Frequency (%)** |
| Otolaryngologists | | 56 (58.95) |
| Pediatric Audiologists | | 28 (29.47) |
| Genetic counselors | | 5 (5.26) |
| Pediatricians | | 4 (4.21) |
| Clinical geneticists | | 2 (2.11) |

**Supplementary Table 1: Provider Survey Results**

**Supplementary Table 2: Parent Recall of Child Hearing Loss Type**

| **Type of Hearing Loss in Child as Reported by Parent** | **Right Ear** | **Left Ear** |
| --- | --- | --- |
| Cause of Hearing Loss | **Frequency (%)** | **Frequency (%)** |
| Sensorineural (inner ear and nerve) | 19 (73.08) | 19 (73.08) |
| Conductive (outer ear) | 4 (15.38) | 4 (15.38) |
| Mixed | 1 (3.85) | 2 (7.69) |
| Auditory Neuropathy | 2 (7.69) | 1 (3.85) |

**Supplementary Table 3: Parent Knowledge Measure**

Summary of Correct Answers by Question (n=38)

(n=38, one survey participant was excluded based upon not answering the questions)

| **Question** | **Frequency (%) Correct** |
| --- | --- |
| 1. Some disease are caused by genes, environment, and lifestyle. (n = 38) | 37 (97.37) |
| 2. A gene is a disease. (n = 37) | 35 (94.59) |
| 3. You can see a gene with the naked eye. (n = 37) | 35 (94.59) |
| 4. Healthy parents can have a child with an inherited disease. (n = 38) | 37 (97.37) |
| 5. A person with an altered (mutated) gene may be completely healthy. (n = 37) | 35 (94.59) |
| 6. All serious diseases are inherited. (n = 38) | 37 (97.37) |
| 7. Genes are instruction for making proteins, which help the body grow and work properly. (n - 37) | 34 (91.89) |
| 8. The child of a person with an inherited disease will always have the same disease. (n=37) | 34 (91.89) |
| 9. A gene is a piece of DNA. (n=38) | 35 (92.11) |
| 10. Altered (mutated) genes can cause disease. (n=38) | 34 (89.47) |
| 11. Genes are inside of cells. (n=37) | 34 (91.89) |
| 12. A chromosome contains many genes. (n=38) | 38 (100.00) |
| 13. Genes determine traits such as height, eye color, and facial appearance. (n = 38) | 38 (100.00) |
| 14. A person has thousands of genes. (n = 38) | 38 (100.00) |
| 15. Identical twins have different sets of genes. (n=36) | 20 (55.56) |
| 16. Humans have 20 pairs of chromosomes. (n=37) | 25 (67.57) |
| 17. Parents pass both copies of each chromosome to their child. (n=36) | 22 (61.11) |
| 18. A genetic test can tell you if you have a higher chance to develop a specific disease. (n=36) | 36 (94.74) |

Supplementary Table 4: Summary of Genetic Knowledge Scores by Education Status (n=38).

Education status was borderline significant (p = 0.052) assessed via Mann-Whitney U-test

| Education Status | Mean (SD) | Median [Min, Max] |
| --- | --- | --- |
| Less Than Bachelor's Degree (n=15) | 15.07 (2.12) | 15 [12, 18] |
| Bachelor's Degree or Higher (n=23) | 16.42 (1.38) | 17 [13, 18] |

**Supplementary Table 5: Parent Survey Response: Benefits, Drawbacks, and Interest in Genetics**

| **Benefits of genetic testing discussed** | **Frequency (%)** |
| --- | --- |
| May provide information about the prognosis for my child | 16 (41.03) |
| Can determine if hearing loss in my child is genetic or not | 16 (41.03) |
| May define the chances for me to have another child with hearing loss | 15 (38.46) |
| May define the chances of my child with hearing loss to have a future child with hearing loss | 13 (33.33) |
| May indicate the need for additional medical evaluations by other types of doctors | 12 (30.77) |
| The benefits of genetic testing for hearing loss were not discussed with me | 11 (28.21) |
| May help guide treatment decisions for my child | 9 (23.08) |
| May have psychological benefits by knowing an answer to the cause of hearing loss in my child | 9 (23.08) |
| Other | 1 (2.56) |
| **Drawbacks of genetic testing discussed** | **Frequency (%)** |
| Genetic testing may not provide an answer for the reason my child has hearing loss | 17 (43.59) |
| The cost of genetic testing is very expensive | 15 (38.46) |
| The drawbacks of genetic testing for hearing loss were not discussed with me. | 13 (33.33) |
| A genetic diagnosis will not change the management and treatment plan of my child | 7 (17.95) |
| Genetic testing could cause stress or anxiety | 4 (10.26) |
| Other | 1 (2.56) |
| There is a risk for genetic results to impact my child’s ability to get a job in the future | 0 (0) |
| There is a risk for genetic results to impact my child’s ability to get health insurance | 0 (0) |
| There is a risk for genetic results to impact my child’s ability to get life insurance | 0 (0) |
| **If you had the opportunity, how likely is it that you would pursue a genetics evaluation for your child?** | **Frequency (%)** |
| I am likely/very likely to pursue a genetics evaluation for my child's hearing loss. | 17 (44.73) |
| I am not sure if I would pursue a genetics evaluation for my child's hearing loss. | 8 (21.05) |
| I am unlikely/very unlikely to pursue a genetics evaluation for my child's hearing loss. | 7 (17.89) |
| Not applicable - I already know the genetic cause of my child's hearing loss | 6 (15.79) |

**Supplemental Figure 1: Provider Discussion of Benefits and Drawbacks of Genetic Testing**

**
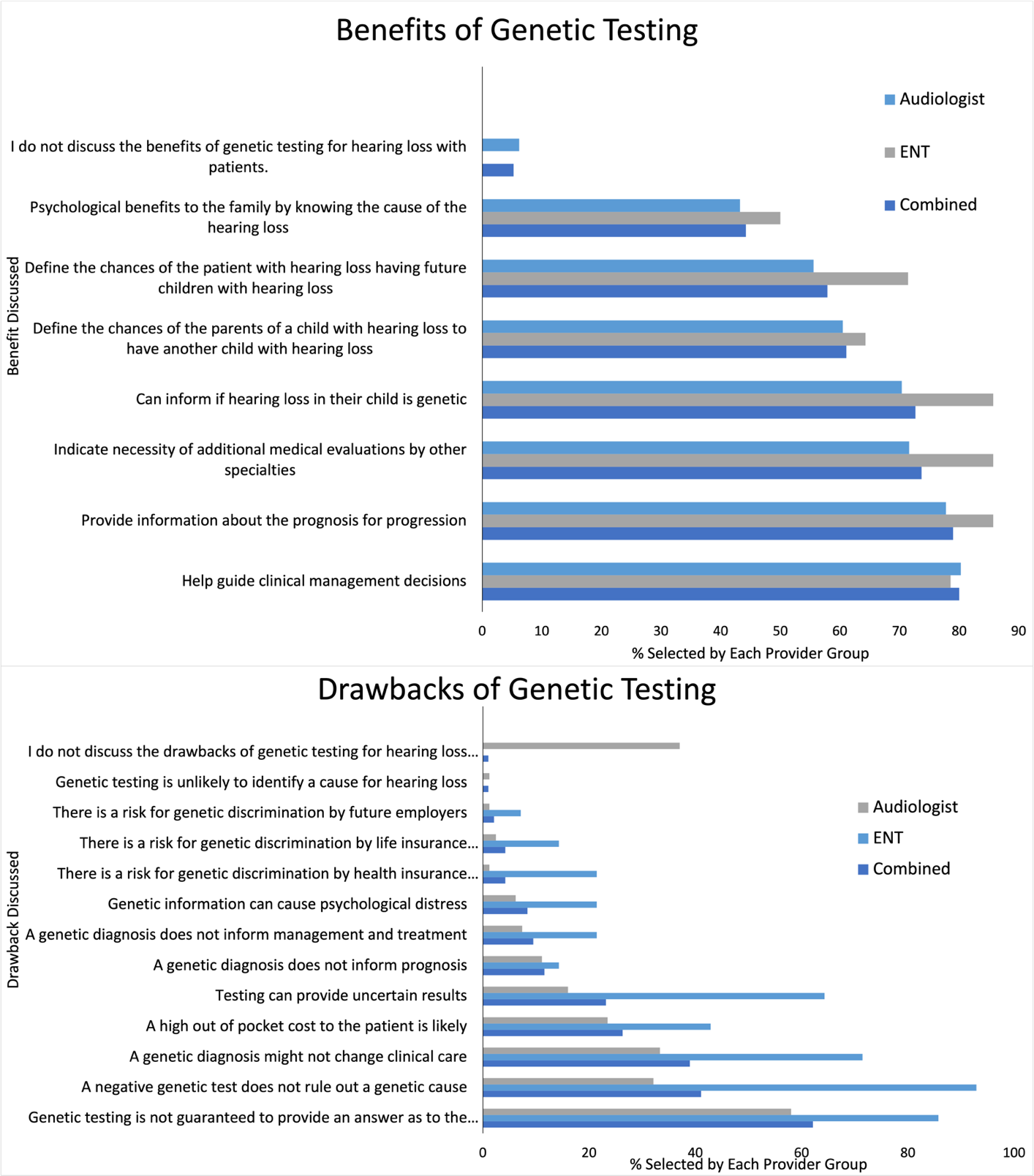
**

**Supplemental Data: Appendix 1: Provider Survey Materials**

**Supplemental Data: Appendix 2: Parent Survey Materials**
